# Utilization of Genotyping-by-Sequencing (GBS) for Rice Pre-Breeding and Improvement: A Review

**DOI:** 10.3390/life12111752

**Published:** 2022-11-01

**Authors:** Vincent Pamugas Reyes, Justine Kipruto Kitony, Shunsaku Nishiuchi, Daigo Makihara, Kazuyuki Doi

**Affiliations:** 1 Graduate School of Bioagricultural Sciences, Nagoya University, Nagoya 464-8601, Japan; 2 International Center for Research and Education in Agriculture, Nagoya University, Nagoya 464-8601, Japan

**Keywords:** molecular breeding, DNA markers, genomic selection, NGS

## Abstract

Molecular markers play a crucial role in the improvement of rice. To benefit from these markers, genotyping is carried out to identify the differences at a specific position in the genome of individuals. The advances in sequencing technologies have led to the development of different genotyping techniques such as genotyping-by-sequencing. Unlike PCR-fragment-based genotyping, genotyping-by-sequencing has enabled the parallel sequencing and genotyping of hundreds of samples in a single run, making it more cost-effective. Currently, GBS is being used in several pre-breeding programs of rice to identify beneficial genes and QTL from different rice genetic resources. In this review, we present the current advances in the utilization of genotyping-by-sequencing for the development of rice pre-breeding materials and the improvement of existing rice cultivars. The challenges and perspectives of using this approach are also highlighted.

## 1. Introduction

Since the completion of the rice genome sequencing in 2005 [1], the identification of agronomically important genes and QTL for rice improvement has been greatly accelerated [2,3,4]. The information from the sequencing of the rice genome resulted in the success of modern breeding practices in rice. However, the current yield performance of the existing rice cultivars may not be sufficient due to the rapid increase in the human population. In addition, intensive rice breeding practices have narrowed the genetic base of elite germplasm. This has compromised the long-term genetic gain and increased the genetic vulnerability to various stresses. Therefore, an efficient method for the identification and incorporation of new genetic variations is necessary.

**Figure 1:**
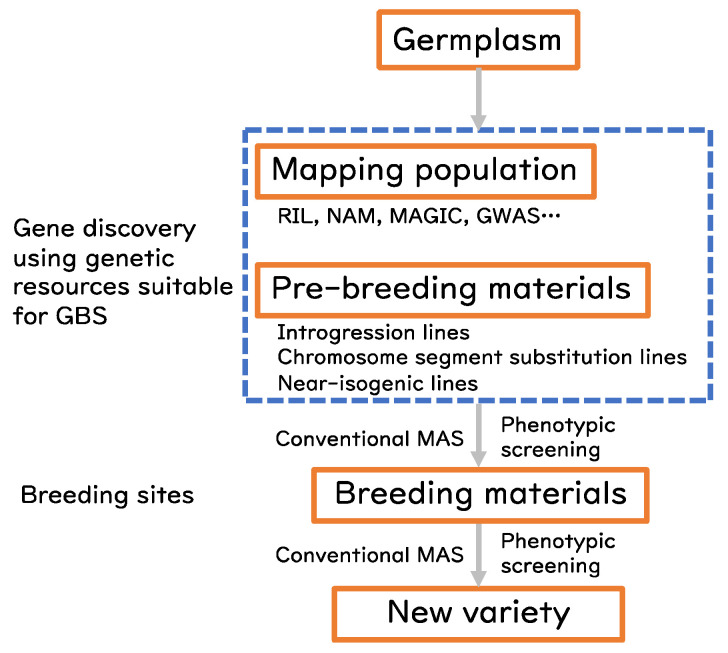
Conventional scheme for gene discovery and breeding. Materials in the hatched blue box are suitable to apply GBS.

Pre-breeding refers to the intermediate step of breeding for rational use of unused landraces, alien germplasm, or wild relatives, which are often not adapted to actual breeding or production sites. The outcome of pre-breeding activities is the development of materials that are suitable for evaluation at actual breeding sites. The materials are often referred to as introgression lines [5] or as chromosome (segment) substitution lines in elite genetic backgrounds. These materials have uniform genetic backgrounds and are well adapted to the breeding sites, thus works as genetic “library” to screen various traits. The traits can be associated with the chromosomal regions defined by DNA markers. In addition to the pre-breeding materials, genetically fixed experimental materials, such as recombinant inbred lines (RILs), nested association mapping (NAM) populations [6,7], multi-parent advanced generation inter-cross (MAGIC) populations [8,9] or genome-wide association (GWA) panels [10,11,12], are publicly available for gene discovery. Fine genotyping of these genetic resources brings a big advantage to efficiently identify the causal genes (Figure 1).

Genomic selection (GS), on the other hand, can be utilized to improve the efficiency in breeding of quantitative traits. Although bi-parental mapping and GWAS approaches were successful in dissecting the genetic architecture of a trait, only a limited proportion of the total genetic variance is explained by the markers. GS offers an alternative concept where favorable genetic variations are targeted across the whole genome without defining the threshold to find significant QTL [13]. In brief, this approach develops a prediction model based on the phenotypic and genotypic information of the training population. This information is then used to establish the genomic estimated breeding values (GEBVs) of all individuals of the breeding population from their genotypic information [14,15]. As compared to QTL-based marker-assisted selection (MAS), GS needs more markers than QTL analysis, hence the need for high throughput-sequencing technologies.

Currently, most of the rice breeding projects are designed by combining phenotypic selection and MAS. At the actual breeding sites, MAS is conducted in early to late generations, depending on the breeding targets. It is supposed that most of the breeders are using PCR-fragment-based markers, such as SSR markers, for foreground selection for a limited number of favorable alleles. On the other hand, background or whole-genome selection is becoming common, but its use is still limited. This is probably because most of the actual breeding materials are discarded, and thus the cost for whole genome selection never suits for a program. However, cost-effective genotyping approaches such as genotyping-by-sequencing (GBS) are promising for conducting background selection or GS. In this review, we present the current advances in the utilization of GBS in the pre-breeding and improvement of rice. Challenges and perspectives regarding the utilization of GBS are also presented.

## 2. Advances in Chemistry of GBS

Over the years, sequencing technologies have become a fundamental approach in the analysis of genetic variation. The rapid advancement in these technologies has led to the development of sequencing platforms that yield millions to billions of DNA bases per run, such as Roche 454, Illumina MiSeq and HiSeq, and Ion torrent [16,17,18,19]. This then enabled the identification of single nucleotide polymorphisms (SNPs) within the genome. Additionally, to overcome the drawbacks of hybridization-based and PCR-fragment-based markers, the use of single nucleotide polymorphism (SNP) markers has been adopted in rice genetics research, as advances in chemistry have allowed cost-effective multiplex sequencing.

GBS is a reduced representation sequencing approach that generates thousands of markers at a low cost. As compared to other genotyping techniques, it is more flexible as it is independent of prior genomic information in most cases. To enable different GBS approaches, different chemistries for library construction have been developed. As summarized in Table 1, approaches for GBS can be categorized into three different types: (1) restriction enzyme-based, (2) PCR-based, and (3) target capture. Among these three, restriction-enzyme based GBS has been widely used. However, the digest of sample DNA and ligation to adaptors depends on the quality of DNA, whereas the PCR-based methods can accept small amounts of low-quality DNA, and labor is reduced in library development. Thus, it is more feasible as compared to other methods and will be more popular in the future. On the other hand, the target capture needs preliminary information about the target sites and the design of the probes, making it less cost-effective for breeding.

### 2.1. Restriction Enzyme-Based (RE-Based) GBS

The RE-based GBS employs the use of a restriction enzyme to digest the genomic DNA. This GBS approach is generally categorized into two types: single-enzyme and double-enzyme. The use of the single-enzyme approach for reduced genome complexity was first described by Baird et al. [21] in restriction association DNA sequencing (RAD-seq). In this technique, the restriction enzyme is used to generate genomic fragments (digestion), which are then ligated to a set of unique adapters that enables the pooling of multiple samples (multiplexing). Pooled samples are then size selected (~300–600 bp for Illumina sequencing), amplified by PCR, and then sequenced. In 2011, Elshire et al. [20] simplified the RAD-seq, herein referred to as Elshire’s GBS, by removing random shearing and size selection steps. In Elshire’s GBS, barcoded adapters and common adapters have an overhanging site that matches the restriction sites. These sites are then ligated onto digested fragments in a single sticky end-ligation. As compared to RAD-seq, Elshire’s GBS is less complicated as the generation of restriction fragments is more straightforward.

The double-enzyme approach was introduced by Peterson et al. [23] using the double-digest RAD (ddRAD). Unlike the single-enzyme GBS, this approach capitalizes on the use of two kinds of REs. This provides a greater degree of complexity reduction. The ddRAD shares almost the same library construction steps as the RAD-seq with minor modifications. In ddRAD, the barcoded adapter is ligated to one end and the common adapter is ligated to the other. The random shearing step was also eliminated in ddRAD, but size selection was retained to recover regions that are randomly distributed in the genome. Another double-enzyme approach, the ezRAD, was introduced by Toonen et al. [33]. In this approach, the flexibility to use any restriction enzyme (or combination) that frequently cuts to generate a desired fragment size has been introduced. Unlike ddRAD, the adapters of ezRAD are not custom designed for the preferred enzyme. This allows researchers to try different enzymes without a costly investment for each enzyme of choice. Elshire’s GBS technique was modified by Poland et al. [24] by adopting the two-enzyme approach. In Poland’s GBS, a combination of rare- and common-cutting REs is used to digest the DNA sample. The digested DNA fragments will contain alternate ends which are fitted to the barcode adaptors and the reverse (Y) adaptor. This approach has been demonstrated to capture fragments that are associated with rare-cutting enzymes. In addition, the use of a Y adaptor on the common restriction avoids the amplification of more common fragment; this is a preferential situation for larger and more complex genomes.

To date, the RE-based GBS approaches have been widely used due to their advantage in terms of scalability, which is dependent on the choice and combinations of restriction enzymes used. However, the enzyme of choice can be a major limitation of these approaches, as the genomic distribution of SNPs is dependent on the specific choice and combination of REs.

### 2.2. PCR-Based GBS

Although RE-based GBS approaches have been utilized in marker-assisted studies, the need for high quality and quantities of DNA has become their limiting factor to being widely adopted. To address this issue, Suyama and Matsuki [29] proposed changing the RE-based steps to PCR-based steps. This approach was based on conventional RE-based markers such as restriction fragment length polymorphism (RFLP) and amplified fragment length polymorphism (AFLP), which were later replaced by the PCR-based marker, simple sequence repeat (SSR).

Generally, PCR-based GBS can be categorized as either random or targeted. The random PCR-based GBS was first introduced by Suyama and Matsuki [29] using multiplexed ISSR genotyping by sequencing (MIG-seq). In brief, a two-step PCR is carried out in the development of the library. The first PCR step is conducted to amplify the ISSR regions in the genomic DNA using 12 bp SSR sequences with 2 bp anchor oligos at the 3′ tail. The products from the first PCR are then used as a template for the second PCR step. In the second PCR step (tailed PCR), complimentary sequences for the Illumina sequencing flow cell and indices are added, and PCR products are pooled into a single sequencing library. Like RAD, this method has a size selection (300–800 bp) step, and size selected fragments are then sequenced. In a recent study by Nishimura et al. [34], MIG-seq has been successfully applied in different crops for genetic analysis. It was highlighted that the number of bases sequenced using this method is associated with genome size. Hence, this method is more suited for crops with large genomes such as wheat (~17 Gb). Given this, few loci could be sequenced in plant species with small genomes. However, it is worthy of note that polymorphism is not only dependent on the number of loci but also on the genetic distance between accessions. Therefore, MIG-seq also be used in plant species with small genome as long as the samples have a high nucleotide diversity (*π* > 0.01). Another type of random PCR-based GBS is the genotyping by random amplicon sequencing-direct (GRAS-Di). Similar to MIG-seq, the development of GRAS-Di sequencing libraries consists of two sequential PCR steps and a final purification step. However, in GRAS-Di, amplification at the first PCR step uses a 10 bp Illumina Nextera adaptor plus 3 bp random oligomers at the 3′-end, which allows amplification of loci higher than the MIG-seq [26,27,28]. To date, this method has been only applied in several crop genetic research [27,35,36,37]. For example, Enoki and Takeuchi [28] developed this method and applied it to a population of rice backcross inbred lines (BIL). Over 10,000 SNPs that were uniformly distributed in the genome, were detected with a very low missing rate (~1.5%) in the data. Similarly, Kumawat and Xu [36] used this approach and generated ~4000 markers that were used for QTL mapping of seed size and shape in a soybean RIL population.

Another type of PCR-based GBS is the targeted approach, which was firstly introduced by Campbell et al. [31] in their method, genotyping-in-thousands by sequencing (GT-seq). Later on, a method that has the same concept as GT-seq was introduced by Onda et al. [30], called multiplex PCR targeted amplicon sequencing (MTA-seq). These methods also share the same concept as the two-step PCR of MT-Seq and GRAS-Di. However, preliminary information on the identified SNPs is necessary, which makes it less flexible for species that lacks genomic resources.

Among all the PCR-based GBS, although still in its infancy, GRAS-Di has great potential to be routinely used in breeding for the following reasons: (1) it can be applied to thousands of samples by using primer sets at a relatively low cost; (2) the amplified regions are reproducible, hence suppressing the missing data will not be a problem; and (3) it can be utilized even with a small quantity of DNA (<100 ng).

### 2.3. Target Capture

Target capture has been proposed as an alternative for complete sequencing of a large and complex genome. To date, methods such as exome capture have been developed [32,38]. This method is focused on the coding and regulatory regions of the genome, which are primary interests for functional genomics research [39]. In this method, genomic DNA is hybridized to microarrays that contain oligonucleotides that are complementary to the target sequences, followed by elution and sequencing of hybridized DNA. Over the years, this method has been modified where biotin-labeled oligonucleotides are used and retrieved using streptavidin beads, hence the solution-based hybridization [39,40].

The target capture GBS has been described to be advantageous over PCR-based GBS as oligonucleotides in target capture have less specificity compared to PCR primers. However, several drawbacks were also documented. For example, the efficiency of this method can be reduced for probes that overlap multiple exons as intron sequences prevent “probe:exon” hybridization [39]. This is a major issue for samples that do not have a reference genome. Although this has been proposed in the past few years, a low adoption rate of this approach in breeding programs is seen due to the costliness of the design of probes and library development.

## 3. GBS for Rice Pre-Breeding

Over the years, GBS methods have been used in different rice pre-breeding studies. In fact, these GBS methods were already used in rice complex crosses such as MAGIC and NAM populations [6,8,9]. Bandillo et al. [8] developed four multi-parent populations (Indica MAGIC, MAGIC plus, Japonica MAGIC, and Global MAGIC) and were subjected to GBS. The sequence data that were obtained were used for GWAS which led to the detection of known (*Sub1*, *Xa4*, and *xa5*) and novel QTL [8]. Ogawa et al. [9] developed an improved allele mining approach using the Japan-MAGIC (JAM) population. Using GBS, a total of 16, 345 SNPs were identified and used to predict the haplotype blocks. Similarly, Fragoso et al. [7] also used GBS in the genotyping of rice NAM populations. The genotyping data obtained were successfully used for simple QTL and joint QTL mapping. Arbelaez et al. [41] developed two populations of interspecific introgression lines derived from *O. meridionalis* × *O. sativa* cv Curinga and *O. rufipogon* × *O. sativa* cv. Curinga. The utilization of GBS in their study increased the marker density by over 50-fold and led to the identification of small donor introgressions that may have been missed using a lower density of markers. Similarly, Spindel et al. [42] used GBS to generate 30,984 markers for 176 RILs derived from an Indica × Japonica cross. Using these markers, QTL for leaf width and aluminum tolerance were mapped. In 2018, Furuta et al. [43] modified the GBS method for rice by changing the rare-cutting enzyme *Pst*I to *Kpn*I. As a result, a higher per-site coverage of sequence reads were generated, and more samples were multiplexed in the sequencing library. However, a trade-off in the number of sites per sample was observed, but the read information was still sufficient for certain applications. The modified GBS method of Furuta et al. [43] has been successfully applied for linkage mapping, introgression studies, recurrent genome recovery analysis, and GWAS in rice [6,44,45,46]. In a study conducted by Liang et al. [47], GBS was efficiently utilized to study cadmium (Cd) accumulation in rice by conducting GWAS on 270 Indica rice varieties. A total of 79,545 genetic markers were identified and were used to identify QTL associated with Cd accumulation. In a study by Goto et al. [48], GBS was utilized and obtained a total of 2221 SNPs. These SNPs were used to map QTL regions that are associated with sodium removal ability in rice leaf sheaths. Similarly, Waheed et al. [49] utilized this approach and identified *qWU7* and *qWU1* which are associated to drought response. Other QTL/genes that were identified using GBS approach are presented in Table 2.

The success of the GBS approaches in genetic mapping of complex traits and its application in complex populations such as NAM and MAGIC shows that it has great potential to be implemented across various pre-breeding programs.

## 4. Implementation of GS and GBS for Rice Improvement

GS can be used to predict the performance of progeny and thus enrich the starting pedigrees. Previous simulation and empirical studies have shown that GS selection can speed up crop improvement [53,54]. In rice, several simulations and much empirical research on GS have been conducted [55,56,57,58,59,60,61,62,63]. One of the benefits of integrating GBS to GS programs is that it can effectively use genome-wide molecular markers from GBS. For example, Spindel et al. [59] conducted GS and GWAS using 363 elite breeding lines from the International Rice Research Institute (IRRI). Using GBS, the population was genotyped with 73,147 markers, and used for predicting grain yield, flowering time, and plant height. The authors demonstrated that subsetting the SNP markers from 73,147 to 7142 (approximately 1 SNP for every 0.2 cM) and 73,147 to 13,101 SNPs (approximately 1 SNP for every 0.1 cM) does not have a significant difference in GS models for a given trait or validation season. However, when the markers were lower than 7142, prediction accuracies began to decrease in most traits and models. Collectively, their results showed that using a SNP every 0.2 cM (~10,000 markers) is a sufficient marker density for GS in inbreed rice populations.

**Figure 2:**
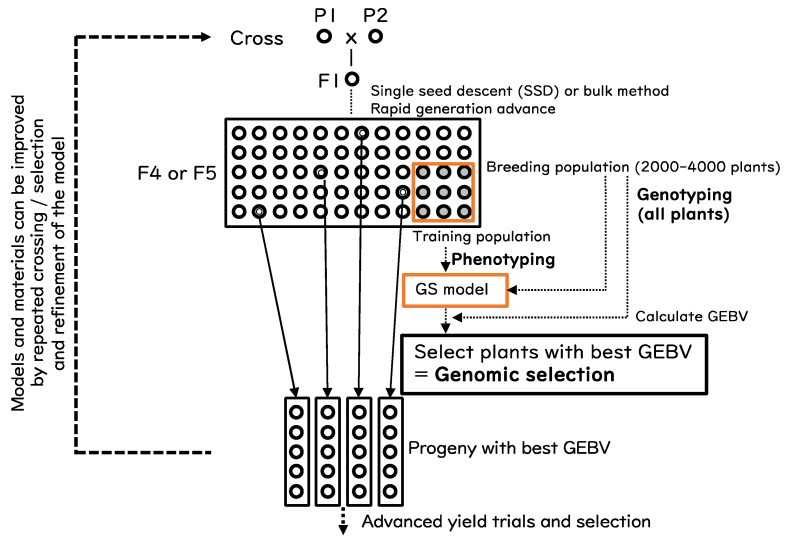
Schematic diagram of a simplified implementation of genomic selection in rice.

A simplified adoption of GS combined with population breeding (bulk method) is proposed in this paper (Figure 2). The breeding population (BP) is generated by single seed descent or by bulk method without selection until F_4_ or F_5_ generation. All plants in BP are genotyped, and a part of BP is subjected to phenotyping to construct the GS model. The GEBV of all plants in BP is calculated and the plants with the best GEBV are selected. The selected progeny are handled in a similar manner to pedigree breeding. If necessary, the GS model can be refined by repeating the whole or a part of the process.

A study by Bassi et al. [64] demonstrated that in wheat, GS in F_3_ or F_4_ generation is the most cost-effective approach with respect to genetic gain and breeding cycles. The same may hold true for rice. We propose to wait until F_4_ or F_5_ to obtain sufficient selection accuracy because GS is most beneficial for polygenic traits. Another important factor is the size of BP and training populations, a larger population provides more possibility to obtain appropriate plants, but this is always limited by external factors such as spaces.

Another viewpoint on the use of GS is to use it for a trait that is not the primary target. In our study using a rice NAM population, more than 90% of the phenotypic variance of heading time can be explained by a model based on genotypes (Kitony et al. unpublished). This means that breeders can enrich the materials by discarding those that do not have the expected heading time. The primary target trait can be selected from the enriched population that have the desired heading time.

## 5. Challenges in Informatics

**Figure 3:**
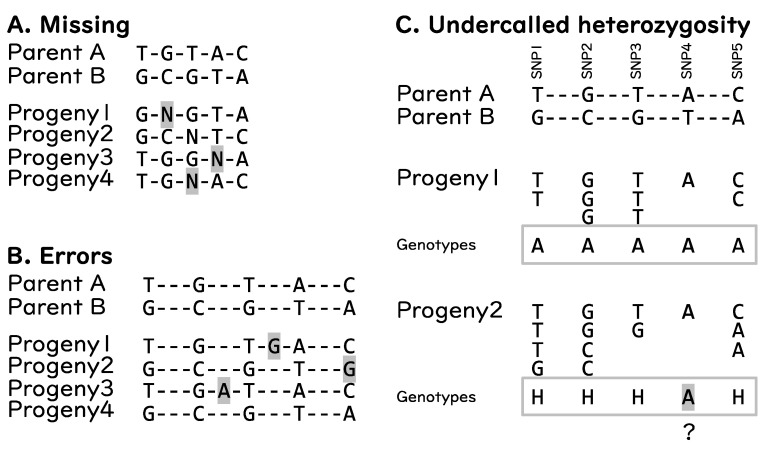
General drawbacks of GBS. (**A**) missing reads resulting in missing genotypes. (**B**) sequencing errors resulting in incorrect genotypes. (**C**) undercalled heterozygosity, SNP4 of progeny 2 is likely to be “H” (heterozygous) but called as “A” (same as parent A). Hyphens (“-”) indicate monomorphic sites. Letters highlighted in gray represent the type of drawback.

Although GBS has become a popular genotyping approach, just like any other tool, this approach has some drawbacks. The major drawbacks associated with GBS are: (1) a large amount of missing data, (2) errors, and (3) undercalled heterozygous genotypes [65] (Figure 3). Missing data in GBS can happen by chance and is primarily due to low coverage sequencing (Figure 3A), whereas sequencing errors are inevitable (Figure 3B). The undercalled heterozygous genotypes result when true genotypes are heterozygous, yet the call is homozygous (Figure 3C). These problems result in wrong calls of genotypes and may affect GS prediction models as they need good molecular datasets [65]. To address these issues, filtering by parents, filtering by minor allele frequency (MAF), and imputation and error correction are necessary.

Sequencing errors can be filtered out by several approaches. Filtering based on minor allele frequency (MAF) < (proportion of 2 different samples against all samples) can be carried out to remove sequencing errors, because if an allele is detected in 2 or more samples, it is likely that the allele is true. Filtering by MAF is not suitable for certain breeding populations such as ILs/CSSLs, but these populations can still be utilized for GBS by using two or more indices for each sample, especially parents. Similarly, filtering by parental genotypes is also an efficient way. This is carried out by selecting SNPs that are (1) not variable in a set of replicated parental samples, (2) non-heterozygous in both parents, and (3) polymorphic between parents.

**Figure 4:**
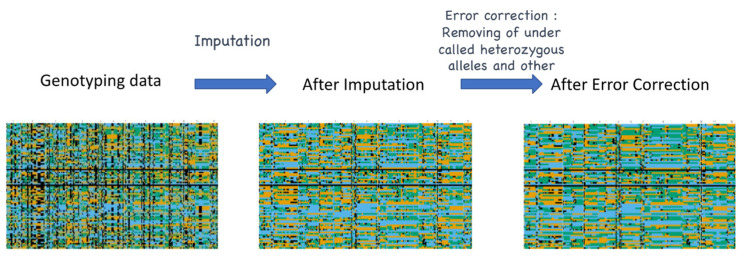
Visual representation of imputation and error correction in GBS data. Black = missing data, Blue = parent 1, Orange = parent 2, Green = heterozygous allele.

For missing data and undercalled heterozygosity, imputation and error correction can be implemented. Imputation refers to a statistical procedure that replaces the missing values in a dataset based on probability. In genotyping, imputation is carried out to predict the untyped loci from the sequencing call and is necessary in any genomic study for a more reliable result [66]. Based on a similar concept, error correction can be conducted. To date, several imputation pipelines have been implemented and evaluated in rice GBS datasets [67,68,69,70]. A comparative analysis by Nazzicari et al. [71] showed that the performance of four general imputation methods (K-nearest neighbors, Random Forest, singular value decomposition, and mean value) and two genotype-specific methods (“Beagle” and “FILLIN”) on rice GBS datasets with up to a 67% missing rate. For general imputation methods, random forest showed the highest accuracy, 90%, whereas Beagle with ordered markers performed well in genotype-specific methods. As a result, the comparison of all methods showed that Beagle with ordered markers outperformed all other imputation methods. An R package called “ABHgenotypeR” for imputation and error-correction on F_2_ populations was previously developed by Furuta et al. [43]. One of the main features of this package is the easy visualization of graphical genotypes for direct comparison (Figure 4). In this analytical tool, imputation is carried out based on the flanking alleles

In this tool, if the genotypes on the left and right of the missing data are identical, then the genotypes are filled in. The package also offers a two-way error correction for GBS datasets: (i) correction of undercalled heterozygous genotypes, and (ii) correction of other genotyping errors. However, in a report presented by Lorieux et al. [72], ABHgenotypeR is not suited for noisy low-coverage sequence datasets. Although several studies have demonstrated the importance of informatics pipelines, error-free GBS dataset is still impossible. However, as demonstrated by Furuta et al. [43] the structural differences between the genomes of the parental lines are more likely to be the major source of the erroneous markers. Therefore, checking the GBS dataset even after the imputation and error correction steps is necessary. For example, manual curation (removal of suspicious SNP calls or markers) of the dataset should be implemented for the outputs of the informatic pipeline.

The high volume of genetic data provided by parallel short-read sequencing also brings serious challenges in the analysis [73]. To fully utilize these GBS datasets, development of software and informatics pipelines that can effectively assemble reads, identify alleles and genotypes, and monitor those genotypes in hundreds of individuals across several populations using a statistically rigorous framework is necessary [74]. In a GBS informatics pipeline, factors such as SNP calling strategies and ease of use must be considered. As summarized in Table 3, Wickland et al. [75] conducted a comparative study on five GBS pipelines.

Among these tools, the GB-eaSy, TASSEL-GBS, and IGST were found to have the highest accuracy (~99%) in terms of SNP calling in comparison with the whole-genome sequence (WGS). Interestingly, a low percentage of common SNPs were detected in these SNP calling tools. Looking at the common SNPs detected using these tools, Wickland et al. [75] only found 12.08% common SNPs between TASSEL, GB-eaSy, and IGST. The difference in the SNPs detected using these tools could be attributed to the SNP calling strategy and read aligners. For example, Hwang et al. [79] conducted a comparison of three read aligners and four variant callers and identified that BWA-MEM together with SAMtools has the greatest accuracy for SNP identification. Similarly, the combination of GB-eaSy with BCFtools/SAMtools showed a great allelic concordance in reference to the WGS data in Soybean lines.

## 6. Future Perspective

The cost reduction in sequencing is allowing a wider application of GBS. In terms of cost-efficiency, multiplexing a greater number of samples for a single GBS run is beneficial for breeders. Several approaches, such as the modification of sequencing adapters, can be carried out to achieve a higher multiplexing capacity. Reyes et al. [80] demonstrated that the addition of indexing reads to barcode adapters of Poland et al. [14] enabled the multiplexing of 2304 samples from independent populations in a single sequencing run. This method improved the convenience of genotyping different populations from multiple breeders.

GBS has enabled the genotyping of thousands of individuals. However, a lack of phenotype information prevents the understanding of polygenic traits because the population is a limiting factor in statistical genetics. Simultaneous utilization of high-throughput phenotyping technologies and GBS will allow a new way of dissecting complex traits. Currently, these new phenotyping technologies are first used for GWAS panels. However, the construction of new genetic resources will greatly accelerate rice breeding.

## Figures and Tables

**Table 1:** Genotyping-by-sequencing methods based on the type of chemistry used.

Approach	Type	Advantages	Disadvantages	Examples	References
Restriction enzyme-based	Single enzyme		Decreased sequencing quality caused by a high proportion of short fragments	RRL, RAD-seq, Elshire’s GBS,	[20,21,22]
	Double enzyme	Provides a greater degree of complexity reduction	Repeatability maybe dependent on size-selection step	ddRAD, Poland’s GBS, SGB, eZRAD	[23,24,25]
PCR-based	Random	Only a small amount of DNA is required High-quality and high-quantity DNA is not necessary Target SNPs can be added flexibly High reproducibility	Optimization is necessary when applied to novel genome	Genotyping by random amplicon sequencing, direct (GRAS-Di); MIG-Seq	[26,27,28]
	Targeted			GT-seq; MTA-Seq;	[29,30,31]
Target capture		Efficiently enrich the target sites	Cost for designing the probes Library development is costly	Exon capture; Capture of known polymorphic sites	[32]

**Table 2:** Detected QTL using the genotyping-by-sequencing technique.

Trait	QTL	Type of Population	Reference
Hybrid weakness	*hwj1* and *hwj2*	F_2_	[45]
Shoot Na^+^ concentration	*qSNC1-1*, *qSNC1-2,* and *qSNC11*	F_2_	[48]
Leaf sheath Na^+^ concentration	*qSHNC1* and *qSHNC11*		
Leaf blade K^+^ -Na^+^ ratio	*qBKNR11*		
Water uptake	*qWU7* and *qWU11*	RIL	[49]
Zinc content in polished rice	*qZPR1.1*	BRIL	[50]
Salinity Tolerance	*qSIS5.1b* and *qSIS6.30*	RIL	[51]
Grain quality	*qGS5.2*, *qGS7.1*, and *qPGWC8*	RIL	[52]

**Table 3:** Comparison of available GBS pipelines.

GBS-Pipeline	SNP Calling Strategy	Ease of Use ^1^	Reference
Trait Analysis by aSSociation, Evolution and Linkage (TASSEL)	Binomial likelihood ratio	Needs extra steps to improve SNP call accuracy. Not built into the pipeline	[68]
IBIS genotyping by sequencing tools (IGST)	Bayesian	-	[76]
Fast-GBS	Haplotype-based	Needs extra steps to improve SNP call accuracy. Not built into the pipeline	[77]
Stacks	Multinomial-based likelihood	-	[78]
GB-eaSy	Bayesian	Additional steps for SNP call accuracy is not needed or built into the pipeline itself.	[75]

^1^ In terms of carrying out all the steps needed to produce accurate SNPs.

## Data Availability

Not applicable.
